# Impact of the COVID-19 Pandemic on Tuberculosis Testing and Treatment at a Tertiary Hospital in Zambia

**DOI:** 10.4269/ajtmh.22-0689

**Published:** 2023-03-13

**Authors:** Joelle I. Rosser, Christabel Phiri, Juliette T. Bramante, Sombo Fwoloshi, Chipepo Kankasa, Patrick Lungu, Raphael Chanda, Peter Chipimo, Lloyd Mulenga, Cassidy W. Claassen, Duncan Chanda

**Affiliations:** 1 Division of Infectious Diseases & Geographic Medicine, Stanford University School of Medicine, Stanford, California;; 2 University of Zambia School of Medicine, Lusaka, Zambia;; 3 University of Washington School of Medicine, Seattle, Washington;; 4 Zambia Ministry of Health, Lusaka, Zambia;; 5 University Teaching Hospital, Lusaka, Zambia;; 6 Zambia National Public Health Institute, Lusaka, Zambia;; 7 Center for International Health, Education, and Biosecurity, University of Maryland School of Medicine, Baltimore, Maryland;; 8 Institute of Human Virology, University of Maryland School of Medicine, Baltimore, Maryland

## Abstract

Globally, tuberculosis (TB) testing and treatment have declined dramatically during the COVID-19 pandemic. We quantified the change in TB visits, testing, and treatment compared with a 12-month pre-pandemic baseline at the national referral hospital’s TB Clinic in Lusaka, Zambia, in the first year of the pandemic. We stratified the results into early and later pandemic periods. In the first 2 months of the pandemic, the mean number of monthly TB clinic visits, prescriptions, and positive TB polymerase chain reaction (PCR) tests decreased as follow: −94.1% (95% CI: −119.4 to −68.8%), −71.4% (95% CI: −80.4 to −62.4%), and −73% (95% CI: −95.5 to −51.3%), respectively. TB testing and treatment counts rebounded in the subsequent 10 months, although the number of prescriptions and TB-PCR tests performed remained significantly lower than pre-pandemic. The COVID-19 pandemic significantly disrupted TB care in Zambia, which could have long-lasting impacts on TB transmission and mortality. Future pandemic preparedness planning should incorporate strategies developed over the course of this pandemic to safeguard consistent, comprehensive TB care.

## INTRODUCTION

Tuberculosis (TB) is a leading cause of infectious disease–associated mortality worldwide, particularly in sub-Saharan Africa.^1^ The COVID-19 pandemic has threatened to reverse progress made on addressing TB over the past decade.^2^^,^^3^ Across the globe, TB services have been disrupted in numerous ways, ranging from fear of going to the hospital, to supply interruptions, to transportation difficulties, and TB testing platforms being redirected for COVID-19 testing.^4^^–^^7^ In the first year of the COVID-19 pandemic, TB services are estimated to have decreased 20% to 40% globally.^8^^,^^9^ This decrease in TB detection and treatment has huge potential to increase transmission and mortality. A 25% case detection decrease over 3 months has been projected to increase TB deaths by 13% worldwide.^10^ Other models estimate that TB mortality could increase up to 20% by 2025 due to COVID-19 disruptions with the biggest impacts due to delays in diagnosis and treatment.^11^

Although the COVID-19 pandemic has universally challenged healthcare systems, the impact of the pandemic on healthcare services has been heterogenous, varying by service type, country, hospital type, location, and pandemic period evaluated. Evaluating the impact of the pandemic on TB services in different healthcare systems, over time, and showing the cascade of interruptions starting from decreased testing to decreased treatment can provide a more complete picture of how this pandemic has affected TB services and therefore how to promote recovery. Although several studies provide global estimates of TB service decreases or model the impact of those decreases worldwide, there is a limited number of studies evaluating more detailed local impacts, especially in low-income environments.

The COVID-19 pandemic was officially recognized and emergency public health programs to combat it initiated in Zambia in March 2020.^12^^–^^15^ The first Zambia Ministry of Health emergency response plan to include not only COVID-19 efforts but also support of essential health services was unveiled on May 21, 2020.^16^ Between March and May 2020, the Zambian government had increasingly stringent mandates around COVID-19 precautions, which peaked in May 2020.^17^ Around that same time, the largest TB clinic in Zambia switched from a policy of discouraging unnecessary clinic visits to initiating a decentralized testing and treatment program.

In this study, we quantify the change in TB visits, testing, and treatment during the first year of the COVID-19 pandemic at University Teaching Hospital (UTH), the national referral hospital in Lusaka, Zambia, with a dedicated TB clinic. We further examine changes in TB service delivery in early versus later stages of the pandemic.

## MATERIALS AND METHODS

We retrospectively reviewed the number of TB diagnostic tests performed, positive tests, prescriptions administered, and clinic visits attended at UTH TB clinic serving adults regardless of HIV status in Lusaka, Zambia, during pre-pandemic and pandemic periods. Monthly aggregated data were collected from the TB clinic, laboratory, and pharmacy at UTH. The number of diagnostic TB polymerase chain reaction (PCR) tests performed and positive results were available as monthly aggregates and are presumed to approximate the number of patients tested on a one-to-one ratio. The TB test positivity was calculated as the number of positive tests divided by the number of tests performed.

We compared the number of tests, prescriptions, and visits between the pre-pandemic and pandemic periods. The *pre-pandemic period* was defined as the 12-month period just before March 2020 (March 1, 2019–February 28, 2020). The *pandemic period* was defined as the 12-month period after March 2020 (April 1, 2020–March 31, 2021). After plotting the data, we observed a difference between the first few months of the pandemic and later months, in line with the evolving pandemic response at the government and hospital level over the first several months of the pandemic. We therefore stratified the pandemic period into the *early pandemic period* to describe the first 2 months after the pandemic appeared in Zambia (April 1, 2020–May 31, 2020) and the *later pandemic period* to describe the following 10 months (July 1, 2020–March 31, 2021). We compared the absolute number each month for all variables of interest between pre-pandemic and pandemic periods using Student’s *t* test to show the mean difference and 95% confidence intervals. We also calculated the percent change in the mean counts each month between the pre-pandemic and pandemic periods. In our sensitivity analysis, we used a paired Student’s *t* test to compare the 12-month pandemic period to the matched 12 months preceding the pandemic (including March 2020 in the pre-pandemic period) to account for any seasonality or pre-pandemic trends.

## RESULTS

**Figure 1.**
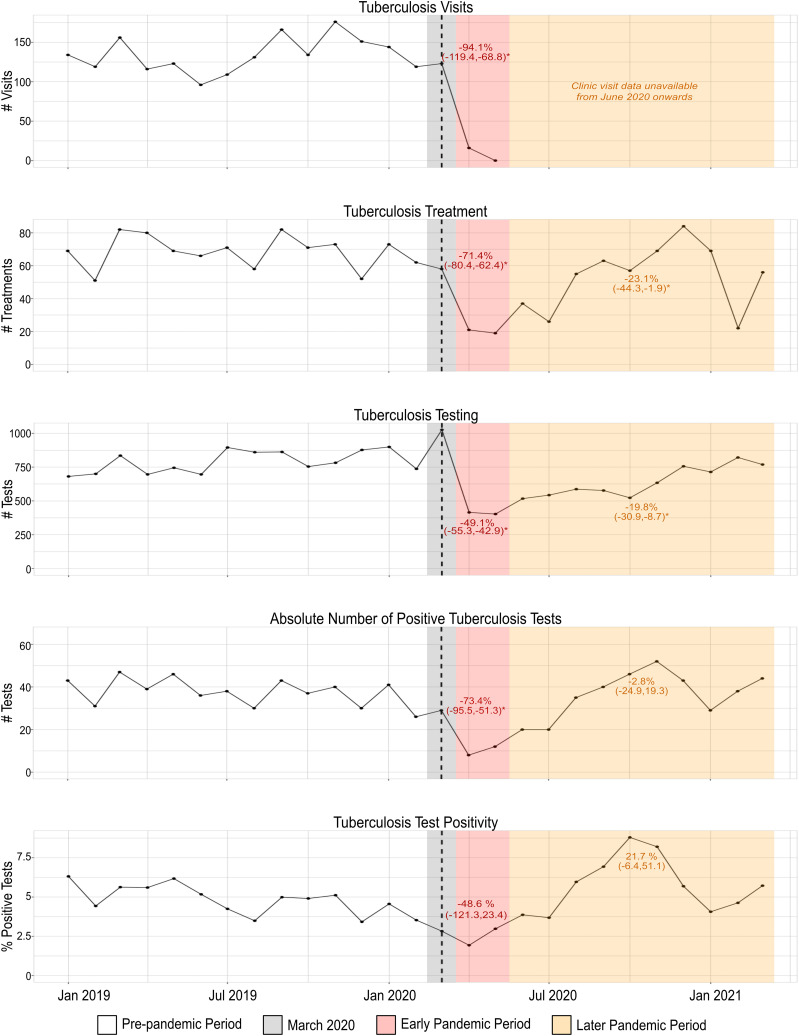
Tuberculosis services decrease precipitously and then rebound during the first year of the COVID-19 pandemic. Dashed vertical lines indicates the start of the COVID-19 pandemic (March 2020). The pink shaded area indicates the early pandemic period (April–May 2020) and the orange shaded area indicates the later pandemic period (June 2020–March 2021). The mean percent change during the early and later pandemic periods compared with the pre-pandemic period are indicated in their shaded areas. *Statistically significant change (*P* < 0.05) from the pre-pandemic period.

During the COVID-19 pandemic, UTH experienced significant decreases in the number of TB clinic visits, TB tests performed, and the number of prescriptions dispensed for TB treatment compared with the year prior (Table 1). These decreases were most dramatic in the first 2 months of the pandemic, with decreases in clinic visits by −94.1% (95% CI: −119.4 to −68.8%), prescriptions by −71.4% (95% CI: −80.4 to −62.4%), and TB tests performed by −49.1% (95% CI: −55.3 to −42.9%) (Figure 1). The subsequent 10 months of the pandemic continued to experience significant, although less dramatic, decreases in TB testing and treatment compared with the pre-pandemic period. After the second month of the pandemic, the TB clinic was shifted to another site, and records of visits numbers were not available.

**Table 1:** Difference in monthly counts of TB services in pre-pandemic vs. early and later pandemic periods

TB service	Pre-PP	PP (12 months)			Early PP (first 2 months)			Later PP (subsequent 10 months)		
	Mean MC	Mean MC	Mean difference (95% CI)	% Change	Mean MC	Mean difference (95% CI)	% Change	Mean MC	Mean difference (95% CI)	% Change
Clinic visits	135.1	–	–	–	8.0	−127.1 (−161.3 to −92.9)*	−94.1 (−119.4 to −68.8)*	–	–	–
Prescriptions	69.9	48.2	−21.8 (−36.6 to −6.9)*	−31.1 (−52.4 to −9.9)*	20.0	−49.9 (−56.2 to −43.6)*	−71.4 (−80.4 to −62.4)*	53.8	−16.1 (−31 to −1.3)*	−23.1 (−44.3 to −1.9)*
TB PCR tests performed	803.2	604.9	−198.2 (−293.5 to −103.0)*	−24.7 (−36.5 to −12.8)*	409.0	−394.2 (−444 to −344.3)*	−49.1 (−55.3 to −42.9)*	644.1	−159.1 (−247.9 to −70.2)*	−19.8 (−30.9 to −8.7)*
Positive TB PCR tests	37.8	32.2	−5.5 (−15.1 to 4.1)	−14.6 (−39.9 to 10.8)	10.0	−27.8 (−36.1 to −19.4)*	−73.4 (−95.5 to −51.3)*	36.7	−1 (−9.4 to 7.3)	−2.8 (−24.9 to 19.3)
Test positivity (%)	4.7	5.2	0.5 (−0.9 to 1.9)	10.0 (−19.1 to 40.4)	2.5	−2.3 (−5.7 to 1.1)	−48.6 (−121.3 to 23.4)	5.8	1 (−0.3 to 2.4)	21.7 (−6.4 to 51.1)

MC = monthly count; PP = pandemic period; TB = tuberculosis. % Change indicates the mean percent change in monthly counts between the pre-pandemic and pandemic period. Data was not available for TB clinic visits after June 2020.

* Statistically significant difference by Student’s *t* test with a *P* < 0.05.

The absolute number of positive TB PCR tests significantly decreased (−73.4%; 95% CI: −95.5 to −51.3%) during the first 2 months of the pandemic; however, this did not hold true in the subsequent 10-month period or when evaluating the first year of the pandemic overall. Additionally, when correcting for the number of tests performed, the TB PCR test positivity was not significantly different during the pandemic versus the pre-pandemic period. These findings were unchanged in sensitivity analyses pairing months during the pre-pandemic and pandemic period, and there was no evidence of pre-pandemic trends or seasonality.

## DISCUSSION

We found significant decreases in TB testing, diagnosis, and treatment during the first year of the COVID-19 pandemic, with the most dramatic decreases experienced during the first 2 months. We also found that TB PCR test positivity, which would not be expected to decrease as a result of the pandemic, showed no significant decrease during this same period and in fact showed a nonsignificant increase in the subsequent 10-month period, perhaps reflective of an increased incidence from delayed diagnoses.

The decreases in TB services that we measured at UTH in Lusaka, Zambia, are consistent with other studies. Our study found a 25% and 31% reduction in TB testing and treatment during the first year of the pandemic, similar to global estimates in TB service reduction by the WHO and Global Fund of 20% to 40%.^8^^,^^9^^,^^18^^,^^19^ We also found that TB PCR testing decreased nearly 50% in the first 2 months of the pandemic, during the early lockdown period, similar to decreases reported in South Africa in the first month of the pandemic (April 2020).^20^

Unlike the many studies that only estimate the overall decrease in services during the first year of the pandemic, our stratified analysis uniquely highlights the dramatically different impact during early versus later phases of the pandemic. The most striking finding in our study is the parallel 73% and 71% decreases in the absolute number of positive TB PCR tests and TB prescriptions in the first 2 months of the pandemic. Multiple models of the long-term impacts of TB service disruption highlight that an accumulation of undiagnosed cases has the biggest impact on TB morbidity and mortality, and that these early shocks can have enormous long-term effects, even with relatively quick recovery to pre-pandemic service levels.^11^^,^^21^ Our approach and findings underline the importance of modeling the long-term impact of TB service disruption as a dynamic, rather than a static, disruption.

Furthermore, by dividing the pandemic periods into early and late, we are able both to see the healthcare system resiliency demonstrated within a few months of the pandemic and to consider the multifaceted ways in which the system can be bolstered for this and future crises. The dramatic decline in the first few months of the pandemic aligns with early lockdown policies by the government and public messaging about limiting nonessential clinic visits. The rebound also parallels the easing of restrictions and TB clinic’s programmatic changes to bolster services. One of the main limitations of this study, the missing data on TB clinic visits after the first 2 months of the pandemic, is in fact due to rapid programmatic adjustments made by the TB clinic to mitigate TB service disruption. In May 2000, the UTH TB clinic started a programmatic decentralization of TB services, encouraging patients to visit their local health centers for TB care. These missing data limit our ability to show whether a rebound occurred in TB clinic visits after its dramatic 94% decrease in the first 2 months of the pandemic. It is also possible that this shift may have been an impediment to accessing services for some individuals and could explain the incomplete rebound in the later pandemic period. On the other hand, this program adaptation highlights an important strategy for continuing high-quality healthcare during a pandemic and the importance of building data collection systems to track the impact of rapid programmatic changes.

Although we intentionally sought to describe the impact in urban Zambia to complement the global estimates of TB service disruption, our study is limited in its ability to characterize the overall differential impact in other, more rural parts of Zambia or to quantify a potential shift in TB service delivery to smaller, local clinics. Our study is also limited in that it is unable to evaluate the reasons behind decreased visits, testing, and treatment; their relative importance; and factors that promoted the rebound in service delivery. Other, qualitative studies early in the pandemic in Zambia indicated that service availability and fear of contracting COVID-19 during clinic visits were major barriers to patients receiving TB services,^5^ and several Ministry of Health and hospital policies were implemented throughout 2020 to bolster TB services.^22^

Despite these limitations, this study provides the first quantitative evaluation of TB service disruption in the largest TB clinic in urban Zambia. Our results clearly demonstrate the immediate shock experienced by TB services at a large urban TB clinic at a tertiary hospital in Lusaka, the relative resiliency of the clinic, and yet the long road ahead to full recovery. Recovery requires more than just a return to baseline but also making up the deficits incurred during the initial shock. Future pandemic preparedness planning should incorporate strategies to immediately transition to decentralized or physically distanced service provision to mitigate the initial health system shock and minimize lasting impacts of testing and treatment interruptions.

## Financial Disclosure

J. I. R. was supported by NIH Training Grant 5T32AI052073-14 and by a Stanford University Center for Innovation in Global Health grant. None of the authors received financial or material support for the research and work in this manuscript.
